# Depression remains a neglected public health problem among pregnant women in Northwest Ethiopia

**DOI:** 10.1186/s13690-021-00649-6

**Published:** 2021-07-12

**Authors:** Getnet Mihretie Beyene, Telake Azale, Kassahun Alemu Gelaye, Tadesse Awoke Ayele

**Affiliations:** 1 Department of psychiatry, College of Health Sciences, Debre Tabor University, Debre Tabor, Ethiopia; 2 Department of Epidemiology and Biostatistics, College of Medicine and Health Sciences, University of Gondar, Gondar, Ethiopia; 3 Department of Health Education and Behavioral Sciences, College of Medicine and Health Sciences, University of Gondar, Gondar, Ethiopia

**Keywords:** Depression, Depressive symptoms, Neglected disease, pregnant, pregnant woman

## Abstract

**Background:**

Antenatal depression is highly prevalent but a neglected public health problem in low income countries. It has serious effects on the general health of women, birth outcomes and child health. However, there has been limited substantial evidence on the prevalence and predictors of antenatal depression in Ethiopia. This lack of evidence potentiates the consequences of the problem and can limit the attention to intervention. Thus, this study aimed to assess the prevalence and potential predictors of antenatal depression at Debre Tabor and Woreta towns, Northeast Ethiopia.

**Methods:**

A community-based cross-sectional study was employed on 548 pregnant women recruited by a cluster sampling method. Depressive symptoms were measured using the Edinburgh Postnatal Depression Scale (EPDS). The List of Threatening Experiences Questionnaire (LTE-Q), the Oslo-3 Social Support Scale (OSSS-3), Intimate Partner Violence (IPV) Scale and Fast Alcohol Screening Test (FAST) were also used to measure stressful events, social support, intimate partner violence (IPV) and hazardous alcohol use respectively. Bivariable and multivariable logistic regression analyses were carried out to identify factors associated with antenatal depression.

**Results:**

The prevalence of antenatal depression was found to be 24.45% (95% CI: 21.20, 28.30%). Being single (AOR =3.32, 95% CI = 1.36, 8.09); fear of pregnancy complication (AOR = 3.84, 95% CI = 1.53,9.62); history of chronic illness (AOR = 8.14, 95% CI = 2.14, 30.91); unplanned pregnancy (AOR = 2.99, 95% CI = 1.36,6.55); history of stillbirth (AOR = 3.56, 95% CI = 1.23, 10.29),one or more negative life events (AOR = 4.06, 95% CI = 1.71, 9.66) and intimate partner violence (AOR = 3.91, 95% CI = 1.65, 9.26) were factors significantly associated with antenatal depression.

**Conclusion:**

Nearly a quarter of pregnant women suffer from depressive symptoms during pregnancy. Being single; fear of pregnancy complication; history of chronic illness; unplanned pregnancy; history of stillbirth; one or more negative life events and intimate partner violence were important predictors of antenatal depression in this study. Health care workers should consider addressing these risk factors during a routine antenatal care. Also, integrating early screening, detection, and treatment of antenatal depression into routine antenatal care is warranted to improve the quality of life of pregnant women and pregnancy outcomes as well.

## Background

Depression is the leading cause of disease burden for women in both high-income and low- and middle-income countries [1–3]. Maternal depression is a neglected public health problem in low- and middle-income countries [4]. Depression is among the most prevalent mental health problems that occur during pregnancy, affecting about one in four women [5].

Evidence from 8% of low- and lower-middle-income countries revealed that the weighted mean prevalence of antenatal common mental disorders was estimated to be 15.6% (95% CI: 15.4–15.9) [6]. The occurrence of depressive symptoms increases considerably in pregnant women than non-pregnant women specifically in mid and late pregnancy [7, 8]. During late pregnancy, fear of childbirth and dysfunctional coping styles are associated with emotional disturbance during late pregnancy [9]. World health organization reported that the prevalence rates of depressive symptoms during pregnancy in low and middle-income countries are estimated to be from 12.5–42% [10].

The prevalence rate of depression during pregnancy in low and middle-income countries is higher than in high-income countries. For example, a comparative study between Pakistani and Canadian women showed that antenatal depression was more prevalent among Pakistani women (48.4%) than Canadian women (31.2% for the Aboriginal and 8.6% for the Caucasians [11]. Reports in several studies carried out in different parts of the world revealed that the prevalence of antenatal depression has huge variation ranging from 3.3% in Sweden to 80% in Pakistan [12, 13].

Various rates of depressive symptoms during pregnancy have been reported from developed and developing countries such as 44.2% in the United States of America [14], 62% in Pakistan [12], 14.2% in Brazil [15] and 39% in South Africa [16].

In Ethiopia, the prevalence estimates of antenatal depression ranges from 6.8 to 32.3% [17–22], by using Edinburgh Postnatal Depression Scale (EPDS) with the cutoff point of ≥13.

Many socio-demographic, clinical, behavioral and psychosocial factors have been reported to be associated with the occurrence and persistence of antenatal depression [19, 20, 23–25]. More frequently reported predictors of depression during pregnancy include low income [11, 16, 26, 27], unplanned pregnancy [6, 15, 19, 20, 23, 27–31], previous history of depression [15, 19, 20, 23, 24, 31], history of stillbirth [20, 23, 32], pregnancy complications [6, 27, 31, 33], intimate partner violence [6, 11, 16, 22, 24, 28, 34, 35], younger age [16, 20, 29], poor social support [16, 20, 22, 26, 30, 31, 33, 35], and stressful events [20, 26, 36]. Pregnancy increases the risk of depression, especially when it is unplanned, has short birth interval, there is financial difficulty and it is out of marriage [4].

In addition to the psychological suffering, untreated maternal depression is associated with adverse health and social consequences for both the mother and the child. Pregnant women with symptoms of depression experience emotional withdrawal and decreased capability to function [37, 38].

Pregnant women with symptoms of depression are more likely to use tobacco, alcohol, and drugs [39]. Moreover, they are subjected to inadequate prenatal care and poorer weight gain in pregnancy, all of which have devastating consequences to the fetus, the baby, and the mother herself [39–41].

Findings showed that untreated prenatal depression is associated with a variety of adverse perinatal outcomes including pre-term birth and low birth weight [42, 43], delayed breastfeeding initiation [44], intrauterine growth restriction [42], admission to neonatal intensive care unit [45] and postpartum depression [12, 41, 46, 47].

Despite the burgeoning evidence on the adverse effects of antenatal depression, the very few published studies in Ethiopia have contradicting findings. Some studies show significant association between antenatal depression and low birth weight [21], prolonged labor, and delayed initiation of breastfeeding [22], pregnancy complications, labor complication and the postpartum complications [23], risk of infant diarrhea [24], increase in non-scheduled antenatal care (ANC) visits and increased number of emergency health care visits [48], child death [25], increased assisted delivery [49], and preterm birth [50, 51].

Although a number of studies have been conducted in Ethiopia, most of them were in other regions with differences in socio-demography, using different tools, and change in time (affects the level of awareness on maternal depression prevention mechanisms) insists us to conduct this study in this area. Therefore, this study aimed to estimate the prevalence and potential predictors of antenatal depressive symptoms among women living at Debre Tabor and Woreta towns of Northwest Ethiopia.

## Methods

### Study design and settings

A community-based cross-sectional study was conducted as part of a prospective cohort study from June to August 2019 in the Northwest, Ethiopia. The study was conducted at Debre Tabor and Woreta towns which are situated in South Gondar zone. According to the South Gondar zone catchment profile, Debre Tabor town has an estimated population of 84,382 of which 40,753 are females and Woreta town has an estimated population of 41,668 of which 20,507 are females. According to the pregnancy rate, about 2844 and 1404 women were estimated to be pregnant per year at Debre Tabor and Woreta towns respectively [52]. In these towns, there were 1 hospital, 5 health centers, and 10 private health institutions providing health services during the data collection period.

At the community level, antenatal care services are carried out by health extension workers who are assigned in each Kebele; the lowest administrative unit or village in Ethiopia. According to the district health office report of the previous year, the proportion of pregnant women who were using antenatal care services at Debre Tabor and Woreta towns were estimated to be 75 and 64% respectively [52].

### Study population, sample size and sampling strategies

The source population consisted of all pregnant women at Debre Tabor and Woreta towns, whereas the study population was all pregnant women in the selected kebeles during the study period. All self- reporting pregnant women in the selected kebeles were eligible for the study, whereas women who were critically ill at the time of data collection were excluded from the study.

The sample size was calculated using a single population proportion formula with a proportion of 11.8% [23], precision of 4, and 95% level of confidence (Standard Normal value of 1.96) the standard normal deviate of 1.96 at 95% confidence intervals. Considering 10% non-response and design effect of 2, the final sample size was 550.

A cluster sampling technique was employed for the selection of the study units. There are ten kebeles in both towns (Debre Tabor Town = 6 and Woreta Town = 4), from which 5 kebeles were selected randomly, by using a lottery method to represent the total population. Participants were identified by obtaining official lists of the pregnant women from health extension workers working in the areas, who routinely collect data on new pregnancies.

### Study variables

The outcome variable in this study was antenatal depression. Depressive symptoms are defined as present when a pregnant woman had an EPDS score of ≥12.

The independent variables were **socioeconomic and demographic characteristics** (maternal age, educational status, marital status, occupation, family monthly income), **obstetric factors** (gravidity, parity,number of live children, fear of pregnancy complication, history of abortion, modes of previous abortion, unplanned pregnancy, gestational age, history of stillbirth, previous pregnancy complication, current pregnancy complication, type of current pregnancy complication, and umber of antenatal service), **Clinical Factors** (previous history of depression and family history of depression, chronic illness and types of chronic illness), **Psychosocial factors** (social support, intimate partner violence, and stressful life events),and b**ehavioral factors** (hazardous alcohol use).

### Measurements

Edinburgh Postnatal Depression Scale (EPDS): We used EPDS to measure depressive symptoms. The EPDS was validated in Ethiopia among postnatal women with a sensitivity and specificity of 78.9 and 75.3% respectively [53], and also has been validated in multiple countries for its use during pregnancy [54–57]. It includes 10 items with a Likert scale of responses scored from 0 to 3, with a maximum score of 30.

Edinburgh Postnatal Depression Scale is more preferable scale than other depression scales to screen depression during pregnancy, because it removes the physical symptoms of depression associated with pregnancy [58].

#### Social support

The Oslo3-item Social Support Scale (OSSS) [59] was used to measure social support. The level of social support is classified as “poor support” 3–8, “moderate support” 9–11 and “strong support”, 12–14 scores. The OSSS-3 contains three items assessing the number of close intimate, perceived level of concern from others and perceived ease of getting helps from neighbors. The OSSS-3 has good convergent and predictive validity [60].

#### List of threatening experiences (LTE)

Experiences of stressful life events during the six months before assessment were assessed using the List of Threatening Experiences (LTE) The scale contains twelve items and includes questions of death, illness, conflicts and loss of property [61]. The presence of stressful life events explained by experienced one or more stressful life events in the last 6 months. LTE has good test-retest reliability (Kappa: 0.61–0.87) and predictive validity [62]. Both the list of threatening experiences (LTE-12) and the Oslo Social Support Scale (OSSS-3) have been used in a population-level study in Ethiopia [63].

#### Intimate partner violence (IPV)

Pregnant women were asked for their exposure to IPV using three questions, one on emotional IPV, one on physical IPV and one on sexual IPV. The presence of IPV was ascertained by the presence of at least one type of IPV [64].

**Fast Alcohol Screening Test (FAST):** is a 4-item self-report measure with 0–4 scores for item 1, 2, 3 whereas 0, 2 and 4 for item 4 and a FAST positive if the total score for all four questions is ≥3 [65].

### Data collection technique and quality control issues

Data were collected by 10 trained data collectors who have experience of data collection and supervised by health officers. Data collectors and supervisors were trained for two days about the study procedures, questionnaires, data collection techniques, quality assurance procedures, and study ethics. Pretest was done at a nearby district on 58 pregnant women to check the clarity of the instrument for independent variables. Based on the finding from the pretest, the questionnaire was revised. The English version of the questionnaire was translated into Amharic and then back into English to maintain its consistency. The collected data were checked daily for completeness and consistency.

### Data analysis

Data were entered, coded, and cleaned using EpiData and exported to STATA software version14 for analysis. Descriptive statistics (frequencies, percentage, means, and standard deviations) were performed.

Bivariate analysis was conducted to assess the relationship between each independent variable and the outcome variable (antenatal depression). To control for the effect of confounding factors, multivariable logistic regression was carried out including variables with a *p*-value of less than 0.2 in the bivariate analysis. The degree of association between dependent and independent variables was assessed using odds ratio with a 95% confidence interval. Hosmer-Lemeshow’s was used to test the goodness-of-fit of the model. To measure the amount of multicollinearity of associated independent variable we use inflation factor (**VIF**). **VIF** and tolerance value were less than 5 and greater than 0.1 respectively, which indicate the independent variables are not linear combinations of each other.

### Ethical issues

Ethical clearance was obtained from the University of Gondar Ethical Review Board and Regional research office. Permission to conduct the study was received from Debre Tabor and Woreta towns’ health department and administration offices.

Written informed consent was obtained from each participant following the provision of an outline of the purpose of the study. Confidentiality was maintained by using a nameless questionnaire and privacy was assured by interviewing the participants alone. Participants who have suicidal ideation were advised to contact psychiatric professionals.

## Results

### Socio-demographic characters of pregnant women

Out of 565 women recruited to participate in the study, 548 agreed (97%), to be part of the study. The mean age of the participants was 27.5 years with (SD, ± 5.3)**,** ranging from 17 to 40 years. The majority of women 499 (91%) were Orthodox Christians by religion. More than a quarter of the participants 148 (27%) attended primary school. Half of the respondents 284 (51.82%) were housewives. From all, 53 (9.7%) women had experienced hunger in the month preceding the interview and 57(10%) had debt to buy food **(**Table **1****).**

**Table 1:** Socio-demographic factors among pregnant women at South Gondar Zone Towns, Northwest Ethiopia, 2020

Characteristics	Frequency (***n*** = 548)	Percent (%)
**Age group**		
≤ 19	30	5.47
20–24	123	22.45
25–29	234	42.70
30–34	91	16.61
> 35	70	12.77
**Religion**		
Orthodox	499	91.06
Muslim	36	6.57
Protestant	13	2.37
**Ethnicity**		
Amhara	534	97.45
Tigre	14	2.55
**Education**		
No education	86	15.69
Primary	148	27.01
Secondary and above	314	57.30
**Occupation**		
Housewife	284	51.82
Employee	126	22.99
Merchant	106	19.34
*Others	32	5.84
**Marital status**		
Single	97	17.70
Married	451	82.30
**Lack of food or Hunger**		
No	495	90.33
Yes	53	9.67
**Debit**		
No	491	89.60
Yes	57	10.40
**Income of family**		
Above the poverty line	441	80.47
Below poverty line	107	19.53

*Others = daily laborer, students

### Obstetric and clinical characteristics

Among the participants who had a history of abortion, 45 (80.36%) and 11 (19.64%) reported spontaneous and induced abortions respectively. History of stillbirth was reported by 37 (11.21%) of participants. The current pregnancy was unplanned for 195 (35.58%) of participating women. One in five, 111 (20.26%) and 47 (8.58%) had a previous history of depression and family history of depression respectively (Table 2).

**Table 2:** Frequency distribution of obstetric and clinical factors among pregnant women at South Gondar Zone Towns, Northwest Ethiopia, 2020

Characteristics	Frequency (***n*** = 548)	Percent (%)
**Depression**		
No	414	24
Yes	134	76
**Number of live children**		
Zero	20	6.06
One	148	44.85
Two-four	154	46.67
Five and above	8	2.42
**previous pregnancy complication(*n*** **= 330)**		
No	255	77.27
Yes	75	22.73
**History of current pregnancy complication**		
No	488	88.14
Yes	65	11.86
**Type of current pregnancy complication (*n*** **= 65)**		
Anemia	23	35.38
APH	13	20
Edema	11	16.92
UTI	18	27.69
**History of abortion(*n*** **= 330)**		
No	274	83.03
Yes	56	16.97
**Modes of previous abortion (*n*** **= 56)**		
Spontaneous	45	80.36
Assisted	11	19.64
**Gravidity**		
One	218	39.78
Two-four	299	54.56
Five and above	31	5.66
**Antenatal service**		
No	57	10.40
Yes	491	89.60
**Number of Antenatal services**		
One	112	22.81
Two	119	24.24
Three	148	30.14
Four	112	22.81
**Previous history of depression**		
No	437	79.74
Yes	111	20.26
**Family history of depression**		
No	501	91.42
Yes	47	8.58
**Parity(*n*** **= 323)**		
One	151	46.75
Two-four	164	50.77
Five and above	8	2.48
**History of stillbirth(*n*** **= 330)**		
No	293	88.79
Yes	37	11.21
**Unplanned Pregnancy**		
Yes	195	35.58
No	353	64.42
**Fear of pregnancy complication**		
No	282	51.46
Yes	266	48.54
**Chronic illness**		
No	503	91.79
Yes	45	8.21
**Type of chronic illness (*n*** **= 45)**		
Anemia	12	26.67
CHF	10	22.22
Hypertension	12	26.67
UTI	11	24.44

### Psychosocial characteristics

Among the participants, 105 (19.16%) reported experiencing one or more stressful life events during the previous six months. Regarding social support, 164 (29.93%) of participants reported poor social support. One hundred five (19.16%) of the participants were misusing alcohol during pregnancy (Table 3).

**Table 3:** Psychosocial, depression and behavioral characteristics of pregnant women at South Gondar Zone Towns, Northwest Ethiopia, 2020

Characteristics	Frequency (***n*** = 548)	Percent (%)
**Life treating events**		
No	443	80.84
Yes	105	19.16
**Social support**		
Strong	147	26.82
Moderate	237	43.25
Poor	164	29.93
**Alcohol misuse**		
No	443	80.84
Yes	105	19.16

### Intimate partner violence

**Fig. 1:**
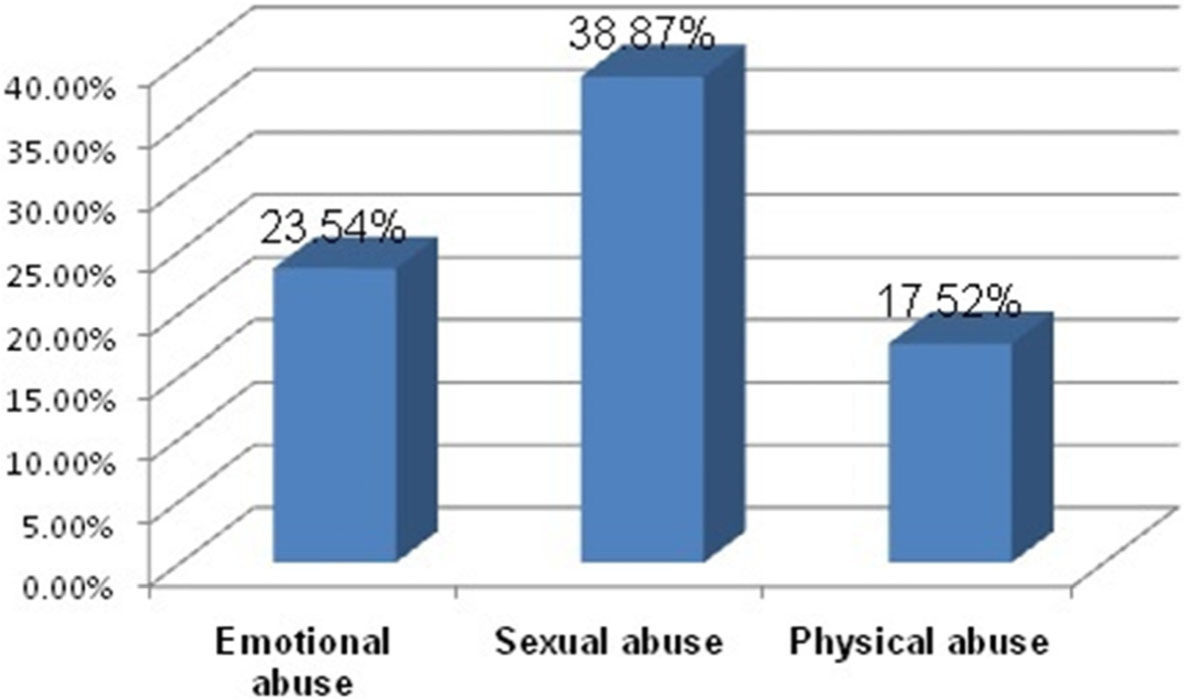
The percentage of intimate partner violence among pregnant women at South Gondar Zone Towns, Northwest Ethiopia, 2020

Among those respondents who experienced intimate partner violence, 38.87% were exposed to sexual abuse (Fig. 1).

### Predictors of antenatal depression

During bivariable analysis, we examined an association between antenatal depression and socio-demographic, obstetric, clinical, psychosocial and behavioral factors and we identified variables at *p* value (P<0.2) to fit into the final model.

Being single (OR = 3.32, 95% CI = 1.36, 8.09); fear of pregnancy complication (OR = 3.84, 95%CI = 1.53, 9.62); history of chronic illness (OR = 8.14,95% CI = 2.14, 30.91); unplanned pregnancy (OR = 2.99, 95% CI = 1.36, 6.55); history of stillbirth (OR = 3.56, 95% CI = 1.23, 10.29), one or more negative life event (OR = 4.06, 95% CI = 1.71, 9.66) and IPV (OR = 3.91, 95% CI = 1.65, 9.26) had significant association with antenatal depression in the multivariable model (Table 4**).**

**Table 4:** Bivariable and multivariable analysis of antenatal depression among pregnant women at South Gondar Zone Towns, Northwest Ethiopia, 2020

Characteristics	Antenatal Depression		COR at 95%CI	AOR at 95%C
	Yes	No		
**Marital status**				
Married	85	366	1	1
Unmarried	49	48	**4.39 (2.767,6.982)**	3.32 (1.363,8.099)**
**Lack of food or Hunger**				
Yes	30	23	**4.90 (2.733, 8.799)**	1.38 (0.112, 17.006)
No	104	391	1	1
**Debit**				
Yes	31	26	**4.49 (2.554, 7.899)**	1.81 (0.164, 20.005)
No	103	388	1	1
**Pregnancy is planned**				
Yes	53	300	**1**	**1**
No	81	114	**4.02 (2.674, 6.048)**	2.99 (1.363,6.547)**
**History of stillbirth(*n*** **= 330)**				
Yes	20	17	**4.47(2.210, 9.059)**	3.56 (1.234, 10.296) **
No	61	232	1	1
**Current pregnancy complication**				
Yes	37	23	**5.26 (3.067, 9.015)**	1.98 (0.653, 5.992)
No	97	391	1	1
**Fear of pregnancy complication**				
Yes	105	161	**5.69 (3.606,8.978)**	3.84 (1.533,9.623)**
No	29	253	1	1
**Chronic illness**				
Yes	23	22	**3.69 (1.984, 6.872)**	8.14 (2.144,30.914) **
No	111	392	1	1
**Previous history of depression**				
Yes	65	46	**7.52 (4.772, 11.900)**	2.04 (0.734, 5.664)
No	69	368	1	1
**Family history of depression**				
Yes	32	15	**8.35 (4.353, 15.997)**	1.65 (0.343,7.942)
No	102	399	1	1
**Negative life event**				
No	67	376	1	1
Yes	67	38	**9.89 (6.151, 15.916)**	4.06 (1.706, 9.659) **
**Social Support**				
Poor	63	101	**2.65 (1.579, 4.451)**	0.74 (0.266, 2.076)
Moderate	43	194	0.94 (0.556, 1.597)	0.68 (0.238, 1.938)
Strong	28	119	1	1
**Intimate partner violence (IPV)**				
Yes	107	118	**9.94 (6.195, 15.953)**	3.91 (1.649, 9.264) **
No	27	296	1	1
**Alcohol misuse**				
Yes	41	64	**2.41 (1.531, 3.796)**	1.08 (0.414, 2.811)
No	93	350	1	1

**N.B:** Abbreviations: **CI** = confidence interval; **OR** = odds ratio; **COR** = Crude odds ratio; **AOR** = Adjusted odds ratio, 1 = Reference, Hosmer-Lemeshow goodness of fit test = 0.7630

** (*P* < 0.01); *(*P* < 0.05)

## Discussion

The prevalence of antenatal depression in this sample was 24.45%(95%CI: 21.20,28.30%). The following factors were associated with increased odds of antenatal depression: Being single, fear of pregnancy complication, history of chronic illness, unplanned pregnancy, history of stillbirth, negative life event and intimate partner violence (IPV).

In the current study, the prevalence of antenatal depression was in line with studies done in the United States of America 25% [66], Canada 27% [67], China 28.5% [68], Brazil 21.2% [25], Nigeria 24.5% [29] and other studies done in Ethiopia such as Addis Ababa 24.94% [19], Hawasa 21.5% [20], Gondar 23% [21], Shashemane 25.6% [22], and Dupti Hospital 17.9% [30].

The result of the present study was higher than the prevalence reported in several other countries such as USA 9% [69], South India 16% [70], Australia 16.9% [26], Brazil 14.2% [15], Bangladesh 18% [24], Portugal 18.5% [71], and at different parts of Ethiopia such as: Gondar town 6.9% [72] and Anended woreda15.20% [28].

The variation in prevalence might be due to methodological differences between studies & study setting (institution vs. community-based). The antenatal depression in this study showed high prevalence because this study was conducted in a population where severely depressed pregnant women were addressed whereas in Portugal, Brazil, US, and Dubti Ethiopia the studies were conducted at health facilities where women with severe symptoms of depression could have remained at home [73]. The other difference might be due the use of different screening tools, cut-off points on screening tools and variation in sample size.

However the result of the present study was lower than the prevalence found in, the United States of America 44.2% [14], Rawalpindi, Pakistan 62% [12] Karachi, Pakistan 81% [74], Korea 40.5–61.4% [75] Cape Town peri-urban settlement, South Africa 39% [16], rural South Africa 47% [76] and other studies in Ethiopia Bale zone 31.5% [77], Sodo district 28.7% [78], and Adama Hospital 31.2% [27].

The prevalence of depression may be influenced by the time point in which symptoms of depression during pregnancy were assessed [79]. In studies such as those in South Africa and Korea recruitment of women at a later stage of pregnancy may lead to the expected burden of delivery and child-rearing might cause a higher rate of depression [80]. Besides this geographic, economic and cultural variations might also contribute to the differences.

A number of variables were found to be significantly associated with depressive symptoms during pregnancy; after adjusting the effects of other demographic, clinical, behavioral and psychosocial variables.

Unmarried women (OR = 3.32, 95% CI = 1.36, 8.09) were 3.3 times more likely to experience antenatal depression as compared to those who were in marriage. The result was consistent with the findings from Nigeria [32, 81]. This might be due to economic hardship, social isolation and parental responsibilities [82].

The results of the present study also revealed that antenatal depression is significantly associated with history of stillbirth. Pregnant women who have a history of stillbirth were 3.56 times more likely to have antenatal depression (OR = 3.56, 95% CI = 1.23, 10.29) than pregnant women who have no history of stillbirth. This was supported by studies conducted in Nigeria [32], in Ethiopia like Debre Tabor [23] and Hawassa [20].

Another predictor identified in this study was unplanned pregnancy. Pregnant women who had not planned their current pregnancy (OR = 2.99, 95% CI = 1.36, 6.55) were 3 times more likely to have antenatal depression than those who planned their pregnancy. This finding is in line with other studies in Ethiopia [19, 20, 23, 27, 28, 83–85], Nigeria [29], Brazil [15], Boston [86], Lithuania [87], and Pakistan [34].

Pregnant women who fear pregnancy complications (OR = 3.84, 95% CI = 1.53, 9.62) were almost four times more likely to experience depression during pregnancy than those without this history. This finding is consistent with a study done in Ethiopia [27]. Women’s fears associated with childbirth are pain, obstetric injury, fear of delivering a physically damaged or congenitally malformed child that might lead to psychological disturbance [88, 89].

Intimate partner violence (IPV) is considered important public health and human rights issue [17]. The current study revealed that intimate partner violence was strongly associated with antenatal depression. In Ethiopia, this association is a concern because domestic violence against women is highly prevalent [90], and especially in populations where low socio-economic status prevails.

The pregnant women who have experienced intimate partner violence (OR = 3.91, 95% CI = 1.65, 9.26) were around four times more likely to experience depression during pregnancy than those without this history. This finding is consistent with a study done in Hyderabad, Pakistan [34], Bangladesh [24], Pakistan, Caucasian and Aboriginal [11], Ethiopia [17, 22, 28, 91], Cape Town, South Africa [16], UK [92], Bangladesh [93], and Tanzania [94].

Negative life events (OR = 4.06, 95% CI = 1.71, 9.66) were significantly associated with depressive symptoms. The result was similar to the study findings in Hawassa, Ethiopia [20, 23], Brazil [15, 25], USA [95], UK [96], and Tanzania [97].

The results indicated that serious chronic illness (OR = 8.14, 95% CI = 2.14, 30.91) was by far the most powerful predictor of depressive symptoms during pregnancy, which increases the risk of depression during pregnancy by eight-fold. This is similar to findings observed in some studies in Canada [11] South Africa [76] Nigeria [29], and Brazil [98]. Depression is one of the most common complications of chronic illness. The illness may limit a person’s ability to interact with others. These changes can be stressful and cause a certain amount of hopelessness or sadness [99].

Since the design is a cross-sectional study, the timing of exposures and the onset of depressive symptoms is not established for every exposure; in particular, chronic illness and depression but only to determine associations. Despite these limitations, the results of this study have significant implication. As this study confirmed that antenatal depression is high and a public health problem; health care providers and policy makers should think about routine screening of all pregnant women for depressive symptoms and to treat them at the primary health care level by integrating the service, since screening is an effective approach for plunging morbidity in depressed people [100]. The EPDS is an easy, quick screening tool to identify women with depressive symptoms, so that, it can be integrated as a part of antenatal care measurement in all health facilities. Antenatal education program and creating awareness about depressive symptoms and its associated factors, adverse effects associated with untreated depression, should be implemented at community and antenatal care levels. It will alert us early screening and treatment of depression symptoms during pregnancy, may help to reduce both severity of depressive symptoms and prevent the adverse effects of depression on neonatal outcomes; moreover, it helps as a standing point for future research.

## Conclusion

Nearly a quarter of pregnant women suffer from depressive symptoms during pregnancy. Being single; fear of pregnancy complication; history of chronic illness; unplanned pregnancy; history of stillbirth; one or more negative life events and intimate partner violence were important predictors of antenatal depression in this study. Health care workers consider to address these risk factors during a rutine antenatal care. Also, integrating early screening, detection, and treatment of antenatal depression into routine antenatal care is waranted to improve the quality of life of pregnant women and pregnancy outcomes as well.

## Data Availability

No additional data is available for this study; all the data are included in the manuscript.

## Abbreviations

ANC: Ante Natal Care
AOR: Adjusted Odds Ratio
CI: Confidence Interval
COR: Crude odds ratio
CM: Common Mental Disorder
EPDS: Edinburgh Postnatal Depression Scale
FAST: Fast Alcohol Screening Test
IPV: Intimate Partner Violence
LTE: List of Threatening Experiences
OR: Odds Ratio
OSSS-3: Oslo-3 Social Support Scale
